# High prevalence and genetic heterogeneity of adenoviruses at a psittacine breeding facility

**DOI:** 10.1007/s11259-024-10533-7

**Published:** 2024-09-12

**Authors:** Gabriele Lizzi, Simone Fasana, Guido Grilli, Giulia Quaglia, Sara Pedrazzoli, Giulia Graziosi, Elena Catelli, Laura Musa, Maria Cristina Rapi, Caterina Lupini

**Affiliations:** 1 Department of Veterinary Medical Sciences, University of Bologna, Via Tolara di Sopra 50, Ozzano dell’Emilia (BO), 40064 Italy; 2 Department of Veterinary Medicine and Animal Science, University of Milan, Via dell’Università 6, Lodi, 26900 Italy

**Keywords:** Psittacine, Adenovirus, *Pol* gene, Molecular characterization

## Abstract

A polymerase chain reaction (PCR) survey was performed at an amateur parrot breeding facility in Italy to investigate the presence and molecular characteristics of adenoviruses. Eighty psittacine birds, belonging to seven parrot species, were sampled by cloacal swabs; in addition, 15 livers were collected from specimens that were found dead. Seventy-two out of 95 samples collected were positive for adenoviruses, with a prevalence rate of 75.8%. All seven psittacine species tested positive for at least one genus of the family *Adenoviridae*; notably, adenoviral infection was found for the first time in the hooded parrot (*Psephotellus dissimilis*). Based on the sequences and phylogenetic analysis, 57 sequences were psittacine adenovirus 2, seven sequences were duck adenovirus 1 and two sequences were identified as psittacine adenovirus 5. The six remaining sequences showed low nucleotide and amino acid identity with the reference strains of accepted species or types, revealing the presence of novel adenoviruses belonging to the genera *Aviadenovirus*, *Barthadenovirus* and *Siadenovirus*. There were identical adenovirus sequences in both apparently healthy and dead birds suggesting that further investigation into the role and impact of these viruses on the health of psittacine birds is warranted.

**Supplementary Information:**

The online version contains supplementary material available at 10.1007/s11259-024-10533-7.

## Introduction

Psittacine birds are affected by many infectious diseases. In particular, viral infections are the most common clinical problems found in captive psittacine species due to their association with sudden death and difficulties in treatment and control (Katoh et al. 2010).

Among these viruses there are those belonging to the family *Adenoviridae*. Adenoviruses (AdVs) are characterised by non-enveloped icosahedral nucleocapsids containing a linear, double-stranded DNA genome that is 25–48 kilobases in length (Benkő et al. 2022). Members of this family are allocated into six genera: *Aviadenovirus*, *Barthadenovirus* (formerly named *Atadenovirus*), *Ichtadenovirus*, *Mastadenovirus*, *Siadenovirus* and *Testadenovirus*, but only *Aviadenovirus*, *Barthadenovirus* and *Siadenovirus* members have been reported in birds. Members of these three genera are widespread among poultry and wild birds throughout the world (Fitzgerald 2020). Infections with AdVs or adenovirus-like particles have been reported from many *Psittaciformes* including genera: *Agapornis* (Jacobson et al. 1989), *Alisterus* (Zadravec et al. 2022), *Amazona* (Hulbert et al. 2015; Lüschow et al. 2007; Zadravec et al. 2022), *Aprosmictus* (Zadravec et al. 2022), *Ara* (Hulbert et al. 2015), *Aratinga* (Gottdenker et al. 2019; Harrach et al. 2023), *Cacatua* (Lüschow et al. 2007; Vaz et al. 2020; Wellehan et al. 2009; Zadravec et al. 2022), *Cyanoramphus* (Konicek et al. 2022), *Eclectus* (Hulbert et al. 2015), *Eolophus* (Vaz et al. 2020), *Melopsittacus* (Hulbert et al. 2015; Katoh et al. 2009; Mori et al. 1989; Zadravec et al. 2022), *Neophema* (Athukorala et al. 2021; Phalen et al. 2019; Yang et al. 2019; Zadravec et al. 2022), *Neopsephotus* (Phalen et al. 2019), *Nymphycus* (Athukorala et al. 2021; Hulbert et al. 2015; Zadravec et al. 2022), *Platycercus* (Mori et al. 1989; Zadravec et al. 2022), *Poicephalus* (Lüschow et al. 2007), *Psittacula* (Lüschow et al. 2007; Wellehan et al. 2009; Zadravec et al. 2022), *Psittacara* (Duarte et al. 2019) *Psittacus* (Zadravec et al. 2022) and *Trichoglossus* (Vaz et al. 2020).

Some avian AdVs are primary pathogens in their own right – for example, turkey haemorrhagic enteritis virus (Rautenschlein et al. 2020), quail bronchitis virus (Reed and Jack 2020) and egg drop syndrome virus or EDSV (Hess et al. 1997; Smyth 2020). Others, however, replicate in birds and cause predominantly subclinical infections with no overt signs of disease until additional factors, such as stress or co-infections with other immunosuppressive viruses, reactivate AdVs with resulting clinical manifestations (Mori et al. 1989; Phalen et al. 2019; Yang et al. 2019). It has been shown that in psittacine birds most AdV infections are subclinical (Athukorala et al. 2021) or cause non-specific symptoms – such as depression and anorexia (Hulbert et al. 2015) or a range of lesions to internal organs – like hepatitis, splenitis, pancreatitis, enteritis, nephritis, conjunctivitis and pneumonia (Wellehan et al. 2009; Yang et al. 2019).

One of the greatest risks of captive bird breeding is the introduction of infections into private collections due to the inclusion of new individuals from other breeding facilities and the common practice to house unrelated species in mixed aviary collections (Phalen et al. 2019).

In the present study, detection and partial molecular characterization were performed at an amateur psittacine breeding facility in Italy. The research was carried out following the death of a hooded parrot (*Psephotellus dissimilis*, previously known as *Psephotus dissimilis*) during the quarantine period, prior to housing. The bird showed diarrhoea and melena. Necropsy revealed haemorrhagic enteritis of the duodenal loop, and histological examination of the affected organs showed the presence of intra-nuclear inclusion body, raising a suspicion of AdV-related disease with multi-organ involvement and irreversible damage.

## Materials and methods

### Psittacine breeding facility and sample collection

The study was conducted at an amateur psittacine breeding facility located in Northern Italy (province of Como, Lombardy region). At the time of sampling, 80 parrots were housed in pairs in cages or aviaries of various sizes. Half of these birds were kept in an outdoor space, both during winter and summer, while the other half were kept in an enclosed space with access to the outside. Occasionally there were small colonies of post-weaning fledglings. The facility does not purchase external individuals except in cases of necessity and any new individuals are introduced into the group after a 30-day quarantine. The seven species reared were: rosy-faced lovebird (*Agapornis roseicollis*), red-crowned parakeet (*Cyanoramphus novaezelandiae*), Bourke’s parrot (*Neopsephotus bourkii*), cockatiel (*Nymphicus hollandicus*), hooded parrot (*Psephotellus dissimilis*), red-rumped parrot (*Psephotus haematonotus*) and rose-ringed parakeet (*Psittacula krameri*).

All living psittacine birds (*n* = 80) were sampled by cloacal swab in November 2022. In addition, 15 livers were collected from specimens found dead from January to August 2022. The details of the samples collected are presented in Table Supplementary 1. The samples were stored at -22 °C ± 3 °C until processing.

### Sample processing and DNA extraction

DNA was extracted using the commercial NucleoSpin^®^ Tissue kit (MACHEREY-NAGEL GmbH & Co. KG, Düren, Germany) according to the manufacturer’s instructions. Cloacal swabs were processed individually by soaking in 400 µl of sterile phosphate-buffered saline, pH 7.4. Livers were individually homogenised and 25 mg were taken for the extraction. DNA was stored at -22 °C ± 3 °C until analysis.

### Nested PCR for AdVs and sequencing

The presence of AdVs was tested by a nested PCR, published previously (Wellehan et al. 2004). This method works with highly degenerate primers that target the most conserved part of the DNA-dependent DNA polymerase gene (*pol*) of AdVs. The sequence of primers for the first run (PCR1) are as follows: forward primer polFouter (5′-TNMGNGGNGGNMGNTGYTAYCC-3′, where N = A, C, G, or T, M = A or C and Y = C or T); reverse primer polRouter (5′-GTDGCRAANSHNCCRTABARNGMRTT-3′, where D = A, G, or T, R = A or G, S = G or C, H = A, T, or C, and B = G, T, or C). For the second run (PCR2) the sequences are: forward primer polFinner (5′-GTNTWYGAYATHTGYGGHATGTAYGC-3′, where W = A or T) and reverse primer polRinner (5′-CCANCCBCDRTTRTGNARNGTRA-3′). In a total reaction volume of 25 µL, 3 µL of DNA (or PCR1 amplicon) was mixed with 0.125 µL of Taq DNA Polymerase (Qiagen), 2.5 µL of CoralLoad PCR Buffer 10x (Qiagen), 2 µL of 25 mM MgCl2 solution (Qiagen), 0.5 µL of dNTPs (0.2 mM – Thermo Fisher Scientific, Massachusetts, MA, USA), 14.875 µL of UltraPure™ Distilled Water DNase/RNase Free (Invitrogen) and 1 µL of each primer (0.2 µM - Sigma). Cycling conditions were the same for the two amplifications: 5 min of denaturation at 94 °C followed by 35 cycles, each consisting of denaturation at 94 °C for 30 s, annealing at 46 °C for 1 min and extension at 72 °C for 1 min (45 s for PCR2). A final elongation step at 72 °C for 5 min completed the reaction. The PCR2 product was separated in 2% agarose gel, stained with MIDORI^green^ Advance (NIPPON Genetics Europe, Düren, Germany), and visualized under ultraviolet light after an electrophoretic run at 110 V and 400 mA for 30 min.

The obtained amplicons were purified using ExoSAP-IT™ Express PCR Product Cleanup Reagent (Thermo Fisher Scientific, Massachusetts, MA, USA) following the manufacturer’s instructions and subsequently Sanger-sequenced by Macrogen Europe (Milan, Italy) using both PCR2 primers.

### Sequence and phylogenetic analysis

The newly obtained nucleotide (nt) sequences were identified using blastn (Basic Local Alignment Search Tool) homology search (Johnson et al. 2008) at the National Center for Biotechnology Information (NCBI) portal. Sequences were assembled and edited using the Bioedit Sequence Alignment Editor, Version 7.2.5.0 (Tom Hall, Ibis Therapeutics, Carlsbad, CA, USA), then aligned and compared using the Clustal W software (Thompson et al. 1994), with psittacine and closest non-psittacine AdV *pol* gene sequences retrieved from the GenBank database (Table Supplementary 2). Phylogenetic trees based on the partial *pol* gene nt and amino acid (aa) sequences were generated using Maximum Likelihood method under, respectively, the Hasegawa-Kishino-Yano model (Hasegawa et al. 1985) and Le_Gascuel_2008 model (Le and Gascuel 2008) in MEGA X (Kumar et al. 2018). The nodal support was calculated by performing 1000 bootstrap replicates; only the nodes of the tree supported by bootstrap values ≥ 70 were considered reliable because they ensure > 95% confidence in the phylogenetic data obtained (Hillis and Bull 1993). A pairwise aa sequence identity analysis was obtained using the Sequence Demarcation Tool, Version 1.3 (Muhire et al. 2014).

### Accession numbers

The obtained sequences were named using the following nomenclature: adenovirus type/country of origin (Italy)/host species/sample ID number/year of detection. Sequences were submitted to the GenBank database and are available under the following accession numbers: PP665606-PP665677.

## Results

### Detection of AdVs at the psittacine breeding facility

The results of the nested PCR conducted on cloacal swabs collected from 80 living psittacine birds are shown in Table 1 and detailed in Table Supplementary 1. The presence of AdV was detected in 63 samples, with a prevalence rate of 78.8%. In particular, 100% (3/3) of the hooded parrots and 92.7% (38/41) of the red-crowned parakeets tested positive. The positivity rate was also high for red-rumped parrots and rosy-faced lovebirds with values of 75% (3/4) and 64.3% (9/14), respectively. Cockatiels and rose-ringed parakeets showed the lowest positivity rate: 58.3% (7/12) and 50% (3/6), respectively.

**Table 1:** Results of nested PCR conducted on cloacal swabs and blastn-based classification

Species sampled	Sampled birds	*N*°	% positivity	AdV type identified			
				*Siadenovirus*			*Barthadenovirus*
				PsAdV-2	PsAdV-5	Not classified	DAdV-1
*Agapornis roseicollis*	14	9	64.3%	6	2	-	1
*Cyanoramphus novaezelandiae*	41	38	92.7%	37	-	-	1
*Nymphicus hollandicus*	12	7	58.3%	5	-	-	2
*Psephotellus dissimilis*	3	3	100%	3	-	-	-
*Psephotus haematonotus*	4	3	75%	1	-	2	-
*Psittacula krameri*	6	3	50%	1	-	-	2
total	80	63	78.8%	53 (84.1%)	2 (3.2%)	2 (3.2%)	6 (9.5%)

The nested PCR results using liver DNA are shown in Table 2. Nine out 15 (60%) of the samples tested positive for *Adenoviridae*. In particular, two Bourke’s parrots tested positive. One of the two rosy-faced lovebirds also tested positive, as did 6 out of the 9 (66.7%) red-crowned parakeets. In contrast, the rose-ringed parakeet and the hooded parrot tested negative for AdV.

**Table 2:** Results of nested PCR conducted on livers and blastn-based classification

Species sampled	Sampled birds	*N*°	% positivity	AdV type identified				
				*Siadenovirus*		*Barthadenovirus*		*Aviadenovirus*
				PsAdV-2	Not classified	DAdV-1	Not classified	Not classified
*Agapornis roseicollis*	2	1	50%	-	-	1	-	-
*Cyanoramphus novaezelandiae*	9	6	66.7%	3	-	-	2	1
*Neopsephotus bourkii*	2	2	100%	1	1	-	-	-
*Psephotellus dissimilis*	1	0	-	-	-	-	-	-
*Psittacula krameri*	1	0	-	-	-	-	-	-
total	15	9	60%	4 (44.5%)	1 (11.1%)	1 (11.1%)	2 (22.2%)	1 (11.1%)

### Sequence analysis

Sequences obtained with the forward and reverse primers were analysed individually and then compared; double peaks were observed in five samples and the highest peak was considered. The nt analysis showed that for 61 of the 63 positive cloacal swabs, it was possible to determine the genus and type (Table 1). In particular, 84.1% (53/63) of the sequences were attributable to psittacine adenovirus 2 (PsAdV-2) of the genus *Siadenovirus*. Additionally, two samples (3.2%) were psittacine adenovirus 5 (PsAdV-5), also part of the genus *Siadenovirus*. On the other hand, six samples (9.5%) belonged to the genus *Barthadenovirus*, specifically to duck adenovirus 1 (DAdV-1). It was not possible to classify the remaining samples taken from two red-rumped parrots (AdV/Italy/*Psephotus_haematonotus*/2026-77/2023 and AdV/Italy/*Psephotus_haematonotus*/2026-78/2023) due to their low nucleotide identity (nt id) and amino acid identity (aa id) with the AdV sequences published in the GenBank database (with a maximum of 77.3% nt id and 91.5% aa id with the BrdKdnyDNA strain).

Concerning liver samples from psittacine birds, sequence analysis allowed the classification of 5 out of 9 positive samples (Table 2). PsAdV-2 was found in a Bourke’s parrot and in three red-crowned parakeets. The positive sample taken from one of the two rosy-faced lovebirds showed the only presence of DAdV-1 in the sampled livers. Four sequences could not be classified: AdV/Italy/*Neopsephotus_bourkii*/2026-84/2023 – identical to the unclassified sequences found in the cloacal swabs of *Psephotus haematonotus*; AdV/Italy/*Cyanoramphus_novaezelandiae*/2026-94/2023 (with a maximum of 76.9% nt id and 95.7% aa id with the AL32 sequence; Zadravec et al. 2022); and AdV/Italy/*Cyanoramphus_novaezelandiae*/2026-86/2023, AdV/Italy/*Cyanoramphus_novaezelandiae*/2026-87/2023 – identical to each other and with a maximum of 71.5% nt id and 68.3% aa id with 22AM sequence; Zadravec et al. 2022.

### Phylogenetic analysis

**Fig. 1:**
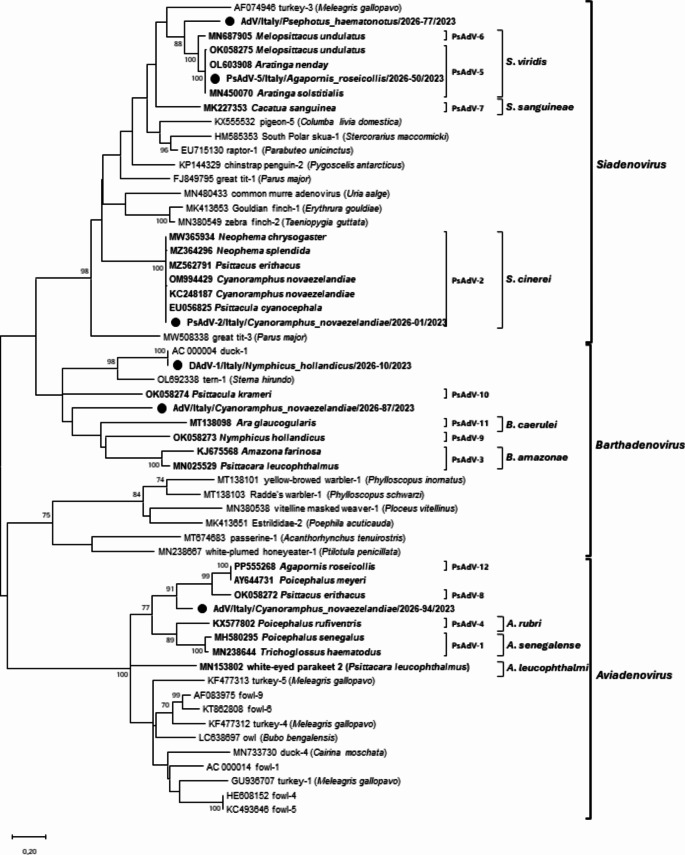
Phylogenetic tree based on the partial pol gene nt sequences of selected AdV strains obtained in this study (marked with a dot), psittacine reference strains (in bold) and non-psittacine reference strains retrieved from GenBank. Since the sequence analysis revealed that the samples of each adenovirus type – detected in our study – had 100% nt id among them, only one sequence per cluster was selected to construct the phylogenetic tree. Psittacine types, species and genera are also indicated on the right of the tree. Only bootstrap values ≥ 70 are shown

**Fig. 2:**
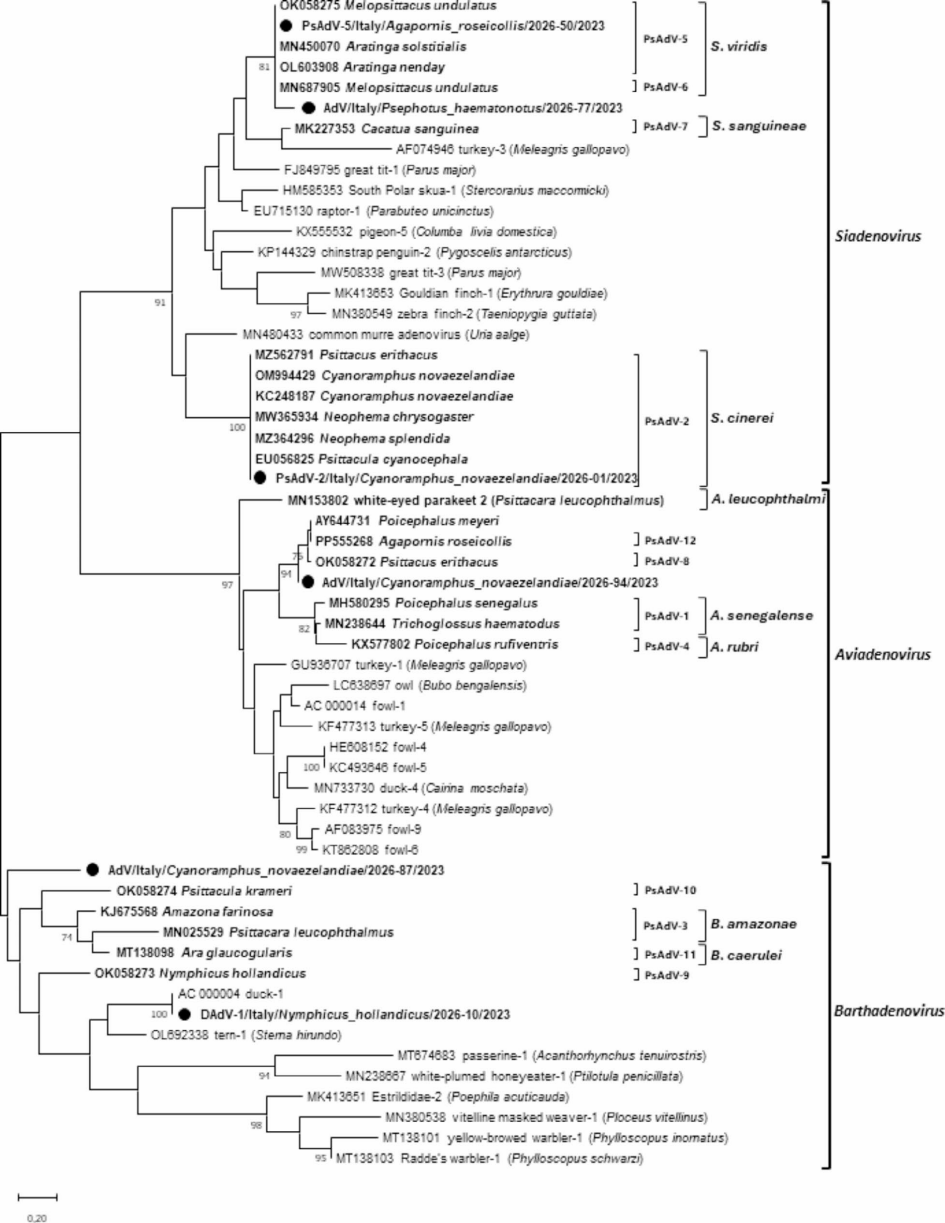
Phylogenetic tree based on the partial pol gene aa sequences of selected AdV strains obtained in this study (marked with a dot), psittacine reference strains (in bold) and non-psittacine reference strains retrieved from GenBank. Since the sequence analysis revealed that the samples of each adenovirus type – detected in our study – had 100% aa id among them, only one sequence per cluster was selected to construct the phylogenetic tree. Psittacine types, species and genera are also indicated on the right of the tree. Only bootstrap values ≥ 70 are shown

Phylogenetic trees based on partial *pol* nt and aa sequences of reference AdV strains and the detected sequences are shown in Figs. 1 and 2, respectively.

Phylogenetic analysis of nt and aa sequences confirmed the classification made by sequence analysis: sequence PsAdV-2/Italy/*Cyanoramphus_novaezelandiae*/2026-01/2023 clustered with the reference PsAdV-2 strains. Sample DAdV-1/Italy/*Nymphicus_hollandicus*/2026-10/2023 clustered with the DAdV-1 reference strain, forming a separate cluster from other barthadenoviruses. Lastly, the analysis confirmed that PsAdV-5/Italy/*Agapornis_roseicollis*/2026-50/2023 belonged to PsAdV-5 type as it clustered with the corresponding reference strains. The unclassified sequences could be assigned to specific genera. Specifically, sequence AdV/Italy/*Psephotus_haematonotus*/2026-77/2023 was classified into the genus *Siadenovirus*. In contrast, sample AdV/Italy/*Cyanoramphus_novaezelandiae*/2026-87/2023 clustered within the genus *Barthadenovirus*, while sample AdV/Italy/*Cyanoramphus_novaezelandiae*/2026-94/2023 formed a distinct branch from other sequences within the genus *Aviadenovirus*.

The pairwise aa sequence id analysis of the partial DNA polymerase aa sequence of unclassified samples, from the closest related reference strain showed that sequence AdV/Italy/*Psephotus_haematonotus*/2026-77/2023 has 8.5% phylogenetic distance with the PsAdV-6 reference strain (BrdKdnyDNA). AdV/Italy/*Cyanoramphus_novaezelandiae*/2026-87/2023 has 31.7% phylogenetic distance from Slovenian sequence 22AM – suggested as psittacine adenovirus 10 (Zadravec et al. 2022). Lastly, AdV/Italy/*Cyanoramphus_novaezelandiae*/2026-94/2023 has 4.3% phylogenetic distance from AL32, another Slovenian sequence suggested as psittacine adenovirus 8 (Zadravec et al. 2022).

## Discussion

The present study reports the circulation of AdVs at a psittacine amateur breeding facility in Italy. The results of the nested PCR revealed a high prevalence of AdV infection: overall 72 of 95 birds tested positive, with a prevalence rate of 75.8%. These data differ from other surveys that reported a lower prevalence. For example, a study conducted among Slovenian living psittacine birds reported an AdV prevalence rate of 16.3% (Zadravec et al. 2022). However, in that case, the sampled birds belonged to different owners and, therefore, a lower prevalence was expected. In Australia, Hulbert et al. (2015) tested 118 psittacine specimens from 11 aviaries but detected only 4 positive sample (3.4%); in their study, the authors applied a PCR targeting the hexon gene with primers designed based on fowl adenovirus sequences, thus making it highly specific compared with the pan-adenovirus primers published by Wellehan et al. (2004) and used in the present study. Conversely, another Australian study corroborates the results obtained in our survey; the research was conducted on orange-bellied parrots bred in captivity at five different institutions and showed a 76.5% AdV prevalence at one of these breeding centres (Yang et al. 2019). In the present study, the cloacal swab sampling method had higher sensitivity: 78.8% of cloacal swabs tested positive compared with 60% of the liver samples. The use of these matrices may seem at odds with the study carried out by Yang et al. (2019), who compared fresh droppings with cloacal swabs to detect AdVs and found that three parrots tested positive only in faeces. In the present study, because the animals were not reared separately, it was more complicated to match fresh droppings to each specimen. On the other hand, sampling birds by cloacal swab reduced the possibility of environmental contamination. Likewise, the choice of analysing liver samples instead of kidney samples is related to the fact that the focus was not on searching for PsAdV-2 – which seems to have tropism for collecting ducts in kidneys (Yang et al. 2019) – but rather for AdVs that are detected most frequently in the liver (Cassmann et al. 2019; Das et al. 2017; Phalen et al. 2019).

In the present study, AdVs were detected in various psittacine species: the rosy-faced lovebird, the red-crowned parakeet, the Bourke’s parrot, the cockatiel, the hooded parrot, the red-rumped parrot and the rose-ringed parakeet. To the best of our knowledge this is the first reported occurrence of AdV infection in the hooded parrot and the first characterisation of AdVs in the red-rumped parrot. Previously, Mori et al. (1989) conducted histopathological examinations on 200 psittacine birds and found intranuclear inclusion bodies (IIB) containing AdV-like particles in 12 of them, including a red-rumped parrot.

In the present study, the red-crowned parakeet was the most sampled species and had a high positivity rate (88%). This prevalence rate is extremely high but it is reflected in the study conducted by Konicek et al. (2022) in which all six parrots, kept in four different households, tested positive for AdVs. Consequently, there may be a high prevalence of AdV infection in this species. Likewise, Bourke’s parrots, hooded parrots and red-rumped parrots showed high positivity rates (100%, 75% and 75% respectively), but the small number of sampled animals does not allow speculation regarding an increased susceptibility of these species to AdV infections. Sixteen lovebirds and 12 cockatiels were tested with 62.5% and 58.3% of birds testing positive, respectively. The result for cockatiels appears to be similar to the survey conducted by Zadravec et al. (2022), who found that 32.7% of the specimens tested positive. On the other hand, the same study showed a lack of positivity among 18 sampled lovebirds. These results highlight the variability of the spread of AdVs among parrot populations and the transmission of AdVs between individuals reared together. Lastly, in the present study the rose-ringed parakeet was the least affected by AdV infection (42.9%).

In our study, the most frequently detected adenovirus type was PsAdV-2, which belongs to the *Siadenovirus* genus: it was found in 79.2% of positive birds. PsAdV-2 was identified in all of the tested species; moreover, based on the available literature, it was found for the first time in rosy-faced lovebirds, hooded parrots and red-rumped parrots. Hooded parrots were the species most affected by the virus – 100% of positive birds – followed by red-crowned parakeets (90.9%) and Bourke’s parrots (66.7%). PsAdV-2 has also been predominant in other studies, representing 66.7% of detected AdVs in Slovenia (Zadravec et al. 2022) or the only psittacine virus isolated among those belonging to the *Adenoviridae* family (Athukorala et al. 2021; Konicek et al. 2022; Phalen et al. 2019; Yang et al. 2019). The second most frequently found AdV in the present study was DAdV-1 (also known as EDSV). This barthadenovirus was detected in 9.7% of the tested birds, including two rosy-faced lovebirds, two red-crowned parakeets, two cockatiels and one rose-ringed parakeet. Although the natural hosts for DAdV-1 are ducks and geese, but a wide range of poultry species such as chickens and quails; waterfowl; and wild birds including gulls, owls and storks, have been shown to be susceptible to EDSV (Smyth 2020). Infection by DAdV-1 has not been reported previously in psittacine birds but, due to the potential ability of AdVs to cross species-specific barriers and switch hosts (Davidson 2021; Phalen et al. 2019) and as well as the high environmental resistance and worldwide spread of EDSV (Smyth 2020), it may have been introduced to this breeding facility and infected the parrots. Lastly, another *Siadenovirus*, PsAdV-5, was detected only in two rosy-faced lovebirds.

Molecular analysis of some of the detected AdVs showed that unclassified sequences had low nt and aa sequence id with reference strains of accepted AdVs species or types, preventing their classification into currently identified types with certainty. Specifically, there was an aviadenovirus type in a red-crowned parakeet, a barthadenovirus type in two red-crowned parakeets and a siadenovirus type in a Bourke’s parrot and in two red-rumped parrots. The species demarcation criterion within each genus of the family *Adenoviridae* is based on the pairwise sequence identity analysis of the complete DNA polymerase aa sequence: > 10-15% distance is required for the demarcation of a new species (Benkő et al. 2022). In the present study, the phylogenetic distances of the unclassified sequences from the closest related reference strain ranged from 4.3 to 31.7% – based on partial DNA polymerase sequences – hinting that they might represent novel AdV types but not always novel species. In particular, the siadenovirus type that we detected might belong to *Siadenovirus viridis* species, together with PsAdV-5 and PsAdV-6 types. Furthermore, the aviadenovirus type that we detected could be a candidate as belonging to a new species not yet proposed, together with PsAdV-8 and psittacine adenovirus 12 types.

The heterogeneity of AdVs observed at the breeding facility highlights how widespread the members of this virus family are and that they may have contributed to the deaths of the parrots; additional studies will be necessary to test this hypothesis. Consistent with the literature (Athukorala et al. 2021; Hulbert et al. 2015; Smyth 2020; Yang et al. 2019), the presence of AdVs is not always correlated with pathogenicity; rather, they can act as a predisposing factor and ‘door opener’ for the entry and exacerbation of other diseases. Only under marginal conditions, following stress or other similar factors, can most AdV infections lead to clinically manifested pathology. Considering that the breeder in the present study does not purchase external individuals – except in case of necessity and then only introduces the new birds into the group after a 30-day quarantine – it would be interesting to evaluate whether the infection was introduced with the purchase of an infected individual that spread the virus, or if the initial group of birds was already infected. One aspect to explore further would be the prevalence of infection in offspring and to determine whether there is the potential for vertical transmission or whether transmission only occurs horizontally.

Due to the high infection rate and heterogeneity of the AdV sequences obtained in this study, and also based on the mixed Sanger chromatograms, it can be assumed that multiple AdV infections might have occurred in some birds. Further analysis, such as Next Generation Sequencing (NGS) or PCR cloning, would be needed to test this hypothesis.

In addition, the well-established presence of groups of free-living parrots in Italy could affect the persistence and spread of AdVs, as they could act as reservoirs of the virus. Therefore, it might also be interesting to assess the incidence of infection in flocks kept in outdoor aviaries compared with those housed indoor, especially in areas where these colonies have established themselves.

Based on the analyses, it is recommended to include AdV detection – using the Wellehan et al. (2004) pan-adenovirus PCR – in screening tests for other transmissible infectious diseases of parrots. This would help to understand the health status of the flocks and to prevent the spread of viruses.

## Conclusions

This study reports the first survey conducted for AdV detection and characterisation from an amateur psittacine breeding facility in Italy. The results showed a high prevalence and heterogeneity of adenoviruses in psittacine birds. Additional studies are required to determine whether the present results are specific to this flock or if they reflect the general condition of other flocks.

All seven psittacine species considered in this study tested positive for at least one genus of the family *Adenoviridae*. Notably, AdV infection was reported for the first time in hooded parrots and this was the first AdV characterisation in red-rumped parrots.

Three unknown AdV types were found, but further analyses are necessary to sequence the entire genome for the possible establishment of new AdV species. Furthermore, because the same AdV types were detected in apparently healthy birds as well as in dead birds, it is necessary to investigate in depth the role and the effects that these viruses have on psittacine health, studying their correlation with other immunosuppressive diseases or predisposing conditions.

## Electronic supplementary material

Below is the link to the electronic supplementary material.

Supplementary Material 1

Supplementary Material 2

## Data Availability

All data used and/or analysed during this study are included in this article and are available from the corresponding author on reasonable request. The sequences generated in this study are available in GenBank^®^ database under accession numbers: PP665606-PP665677.
