# Supplementary material for: High prevalence and genetic heterogeneity of adenoviruses at a psittacine breeding facility
Source: Vet Res Commun. 2024 Sep 12;48(6):4113–22. doi: 10.1007/s11259-024-10533-7 (PMC11538262; doi:10.1007/s11259-024-10533-7)
Supplement: Supplementary file 1 — Supplementary Material 1 [file 11259_2024_10533_MOESM1_ESM.docx]

**High prevalence and genetic heterogeneity of adenoviruses at a psittacine breeding facility**

**Veterinary Research Communications**

Gabriele Lizzi^1^, Simone Fasana^2^, Guido Grilli^2^, Giulia Quaglia^1^, Sara Pedrazzoli^1^, Giulia Graziosi^1^, Elena Catelli^1^, Laura Musa^2^, Maria Cristina Rapi^2^, Caterina Lupini^1^

^1^ Department of Veterinary Medical Sciences, University of Bologna, Via Tolara di Sopra 50, Ozzano dell'Emilia (BO), 40064, Italy

^2^ Department of Veterinary Medicine and Animal Science, University of Milan, Via dell'Università 6, Lodi, 26900, Italy

**Corresponding author:**

Gabriele Lizzi, Department of Veterinary Medical Sciences, University of Bologna, Via Tolara di Sopra 50, Ozzano dell'Emilia (BO), 40064, Italy. Email: gabriele.lizzi2@unibo.it

***Table Supplementary 1****: Details of samples analysed in this study.*

| **Sample ID** | **Species** | **Type of sample** | **Partial *pol* gene nested PCR** | | | **Sequence nomenclature** | **Accession** |
| --- | --- | --- | --- | --- | --- | --- | --- |
|  |  |  | **Result** | **AdV type** | **Genus** |  |  |
| 2026-01/23 | *Cyanoramphus novaezelandiae* | cloacal swab | ***Positive*** | psittacine adenovirus 2 | *Siadenovirus* | PsAdV-2/Italy/*Cyanoramphus_novaezelandiae*/2026-01/2023 | PP665616 |
| 2026-02/23 | *Cyanoramphus novaezelandiae* | cloacal swab | ***Positive*** | psittacine adenovirus 2 | *Siadenovirus* | PsAdV-2/Italy/*Cyanoramphus_novaezelandiae*/2026-02/2023 | PP665617 |
| 2026-03/23 | *Cyanoramphus novaezelandiae* | cloacal swab | ***Positive*** | psittacine adenovirus 2 | *Siadenovirus* | PsAdV-2/Italy/*Cyanoramphus_novaezelandiae*/2026-03/2023 | PP665618 |
| 2026-04/23 | *Cyanoramphus novaezelandiae* | cloacal swab | ***Positive*** | psittacine adenovirus 2 | *Siadenovirus* | PsAdV-2/Italy/*Cyanoramphus_novaezelandiae*/2026-04/2023 | PP665619 |
| 2026-05/23 | *Cyanoramphus novaezelandiae* | cloacal swab | ***Positive*** | psittacine adenovirus 2 | *Siadenovirus* | PsAdV-2/Italy/*Cyanoramphus_novaezelandiae*/2026-05/2023 | PP665620 |
| 2026-06/23 | *Cyanoramphus novaezelandiae* | cloacal swab | ***Positive*** | psittacine adenovirus 2 | *Siadenovirus* | PsAdV-2/Italy/*Cyanoramphus_novaezelandiae*/2026-06/2023 | PP665621 |
| 2026-07/23 | *Cyanoramphus novaezelandiae* | cloacal swab | ***Positive*** | psittacine adenovirus 2 | *Siadenovirus* | PsAdV-2/Italy/*Cyanoramphus_novaezelandiae*/2026-07/2023 | PP665622 |
| 2026-08/23 | *Cyanoramphus novaezelandiae* | cloacal swab | ***Positive*** | psittacine adenovirus 2 | *Siadenovirus* | PsAdV-2/Italy/*Cyanoramphus_novaezelandiae*/2026-08/2023 | PP665623 |
| 2026-09/23 | *Nymphicus hollandicus* | cloacal swab | Negative | --- | --- | --- | --- |
| 2026-10/23 | *Nymphicus hollandicus* | cloacal swab | ***Positive*** | duck adenovirus 1 | *Barthadenovirus* | DAdV-1/Italy/*Nymphicus_hollandicus*/2026-10/2023 | PP665606 |
| 2026-11/23 | *Nymphicus hollandicus* | cloacal swab | Negative | --- | --- | --- | --- |
| 2026-12/23 | *Nymphicus hollandicus* | cloacal swab | Negative | --- | --- | --- | --- |
| 2026-13/23 | *Cyanoramphus novaezelandiae* | cloacal swab | ***Positive*** | psittacine adenovirus 2 | *Siadenovirus* | PsAdV-2/Italy/*Cyanoramphus_novaezelandiae*/2026-13/2023 | PP665624 |
| 2026-14/23 | *Cyanoramphus novaezelandiae* | cloacal swab | ***Positive*** | psittacine adenovirus 2 | *Siadenovirus* | PsAdV-2/Italy/*Cyanoramphus_novaezelandiae*/2026-14/2023 | PP665625 |
| 2026-15/23 | *Cyanoramphus novaezelandiae* | cloacal swab | ***Positive*** | psittacine adenovirus 2 | *Siadenovirus* | PsAdV-2/Italy/*Cyanoramphus_novaezelandiae*/2026-15/2023 | PP665626 |
| 2026-16/23 | *Cyanoramphus novaezelandiae* | cloacal swab | ***Positive*** | psittacine adenovirus 2 | *Siadenovirus* | PsAdV-2/Italy/*Cyanoramphus_novaezelandiae*/2026-16/2023 | PP665627 |
| 2026-17/23 | *Cyanoramphus novaezelandiae* | cloacal swab | ***Positive*** | psittacine adenovirus 2 | *Siadenovirus* | PsAdV-2/Italy/*Cyanoramphus_novaezelandiae*/2026-17/2023 | PP665628 |
| 2026-18/23 | *Cyanoramphus novaezelandiae* | cloacal swab | ***Positive*** | psittacine adenovirus 2 | *Siadenovirus* | PsAdV-2/Italy/*Cyanoramphus_novaezelandiae*/2026-18/2023 | PP665629 |
| 2026-19/23 | *Cyanoramphus novaezelandiae* | cloacal swab | ***Positive*** | psittacine adenovirus 2 | *Siadenovirus* | PsAdV-2/Italy/*Cyanoramphus_novaezelandiae*/2026-19/2023 | PP665630 |
| 2026-20/23 | *Nymphicus hollandicus* | cloacal swab | ***Positive*** | duck adenovirus 1 | *Barthadenovirus* | DAdV-1/Italy/*Nymphicus_hollandicus*/2026-20/2023 | PP665607 |
| 2026-21/23 | *Nymphicus hollandicus* | cloacal swab | ***Positive*** | psittacine adenovirus 2 | *Siadenovirus* | PsAdV-2/Italy/*Nymphicus_hollandicus*/2026-21/2023 | PP665631 |
| 2026-22/23 | *Psittacula krameri* | cloacal swab | ***Positive*** | psittacine adenovirus 2 | *Siadenovirus* | PsAdV-2/Italy/*Psittacula_krameri*/2026-22/2023 | PP665632 |
| 2026-23/23 | *Psittacula krameri* | cloacal swab | Negative | --- | --- | --- | --- |
| 2026-24/23 | *Nymphicus hollandicus* | cloacal swab | ***Positive*** | psittacine adenovirus 2 | *Siadenovirus* | PsAdV-2/Italy/*Nymphicus_hollandicus*/2026-24/2023 | PP665633 |
| 2026-25/23 | *Nymphicus hollandicus* | cloacal swab | Negative | --- | --- | --- | --- |
| 2026-26/23 | *Cyanoramphus novaezelandiae* | cloacal swab | ***Positive*** | psittacine adenovirus 2 | *Siadenovirus* | PsAdV-2/Italy/*Cyanoramphus_novaezelandiae*/2026-26/2023 | PP665634 |
| 2026-27/23 | *Nymphicus hollandicus* | cloacal swab | ***Positive*** | psittacine adenovirus 2 | *Siadenovirus* | PsAdV-2/Italy/*Nymphicus_hollandicus*/2026-27/2023 | PP665635 |
| 2026-28/23 | *Nymphicus hollandicus* | cloacal swab | ***Positive*** | psittacine adenovirus 2 | *Siadenovirus* | PsAdV-2/Italy/*Nymphicus_hollandicus*/2026-28/2023 | PP665636 |
| 2026-29/23 | *Nymphicus hollandicus* | cloacal swab | ***Positive*** | psittacine adenovirus 2 | *Siadenovirus* | PsAdV-2/Italy/*Nymphicus_hollandicus*/2026-29/2023 | PP665637 |
| 2026-30/23 | *Nymphicus hollandicus* | cloacal swab | Negative | --- | --- | --- | --- |
| 2026-31/23 | *Psittacula krameri* | cloacal swab | Negative | --- | --- | --- | --- |
| 2026-32/23 | *Psittacula krameri* | cloacal swab | ***Positive*** | duck adenovirus 1 | *Barthadenovirus* | DAdV-1/Italy/*Psittacula_krameri*/2026-32/2023 | PP665608 |
| 2026-33/23 | *Cyanoramphus novaezelandiae* | cloacal swab | ***Positive*** | psittacine adenovirus 2 | *Siadenovirus* | PsAdV-2/Italy/*Cyanoramphus_novaezelandiae*/2026-33/2023 | PP665638 |
| 2026-34/23 | *Cyanoramphus novaezelandiae* | cloacal swab | ***Positive*** | psittacine adenovirus 2 | *Siadenovirus* | PsAdV-2/Italy/*Cyanoramphus_novaezelandiae*/2026-34/2023 | PP665639 |
| 2026-35/23 | *Cyanoramphus novaezelandiae* | cloacal swab | ***Positive*** | psittacine adenovirus 2 | *Siadenovirus* | PsAdV-2/Italy/*Cyanoramphus_novaezelandiae*/2026-35/2023 | PP665640 |
| 2026-36/23 | *Cyanoramphus novaezelandiae* | cloacal swab | ***Positive*** | psittacine adenovirus 2 | *Siadenovirus* | PsAdV-2/Italy/*Cyanoramphus_novaezelandiae*/2026-36/2023 | PP665641 |
| 2026-37/23 | *Cyanoramphus novaezelandiae* | cloacal swab | ***Positive*** | psittacine adenovirus 2 | *Siadenovirus* | PsAdV-2/Italy/*Cyanoramphus_novaezelandiae*/2026-37/2023 | PP665642 |
| 2026-38/23 | *Cyanoramphus novaezelandiae* | cloacal swab | ***Positive*** | psittacine adenovirus 2 | *Siadenovirus* | PsAdV-2/Italy/*Cyanoramphus_novaezelandiae*/2026-38/2023 | PP665643 |
| 2026-39/23 | *Cyanoramphus novaezelandiae* | cloacal swab | ***Positive*** | psittacine adenovirus 2 | *Siadenovirus* | PsAdV-2/Italy/*Cyanoramphus_novaezelandiae*/2026-39/2023 | PP665644 |
| 2026-40/23 | *Agapornis roseicollis* | cloacal swab | ***Positive*** | duck adenovirus 1 | *Barthadenovirus* | DAdV-1/Italy/*Agapornis_roseicollis*/2026-40/2023 | PP665609 |
| 2026-41/23 | *Agapornis roseicollis* | cloacal swab | ***Positive*** | psittacine adenovirus 2 | *Siadenovirus* | PsAdV-2/Italy/*Agapornis_roseicollis*/2026-41/2023 | PP665645 |
| 2026-42/23 | *Agapornis roseicollis* | cloacal swab | Negative | --- | --- | --- | --- |
| 2026-43/23 | *Agapornis roseicollis* | cloacal swab | ***Positive*** | psittacine adenovirus 2 | *Siadenovirus* | PsAdV-2/Italy/*Agapornis_roseicollis*/2026-43/2023 | PP665646 |
| 2026-44/23 | *Agapornis roseicollis* | cloacal swab | ***Positive*** | psittacine adenovirus 2 | *Siadenovirus* | PsAdV-2/Italy/*Agapornis_roseicollis*/2026-44/2023 | PP665647 |
| 2026-45/23 | *Cyanoramphus novaezelandiae* | cloacal swab | ***Positive*** | psittacine adenovirus 2 | *Siadenovirus* | PsAdV-2/Italy/*Cyanoramphus_novaezelandiae*/2026-45/2023 | PP665648 |
| 2026-46/23 | *Cyanoramphus novaezelandiae* | cloacal swab | ***Positive*** | psittacine adenovirus 2 | *Siadenovirus* | PsAdV-2/Italy/*Cyanoramphus_novaezelandiae*/2026-46/2023 | PP665649 |
| 2026-47/23 | *Cyanoramphus novaezelandiae* | cloacal swab | ***Positive*** | psittacine adenovirus 2 | *Siadenovirus* | PsAdV-2/Italy/*Cyanoramphus_novaezelandiae*/2026-47/2023 | PP665650 |
| 2026-48/23 | *Cyanoramphus novaezelandiae* | cloacal swab | Negative | --- | --- | --- | --- |
| 2026-49/23 | *Agapornis roseicollis* | cloacal swab | ***Positive*** | psittacine adenovirus 2 | *Siadenovirus* | PsAdV-2/Italy/*Agapornis_roseicollis*/2026-49/2023 | PP665651 |
| 2026-50/23 | *Agapornis roseicollis* | cloacal swab | ***Positive*** | psittacine adenovirus 5 | *Siadenovirus* | PsAdV-5/Italy/*Agapornis_roseicollis*/2026-50/2023 | PP665673 |
| 2026-51/23 | *Agapornis roseicollis* | cloacal swab | Negative | --- | --- | --- | --- |
| 2026-52/23 | *Agapornis roseicollis* | cloacal swab | Negative | --- | --- | --- | --- |
| 2026-53/23 | *Agapornis roseicollis* | cloacal swab | ***Positive*** | psittacine adenovirus 5 | *Siadenovirus* | PsAdV-5/Italy/*Agapornis_roseicollis*/2026-53/2023 | PP665674 |
| 2026-54/23 | *Agapornis roseicollis* | cloacal swab | ***Positive*** | psittacine adenovirus 2 | *Siadenovirus* | PsAdV-2/Italy/*Agapornis_roseicollis*/2026-54/2023 | PP665652 |
| 2026-55/23 | *Agapornis roseicollis* | cloacal swab | Negative | --- | --- | --- | --- |
| 2026-56/23 | *Agapornis roseicollis* | cloacal swab | Negative | --- | --- | --- | --- |
| 2026-57/23 | *Agapornis roseicollis* | cloacal swab | ***Positive*** | psittacine adenovirus 2 | *Siadenovirus* | PsAdV-2/Italy/*Agapornis_roseicollis*/2026-57/2023 | PP665653 |
| 2026-58/23 | *Psittacula krameri* | cloacal swab | Negative | --- | --- | --- | --- |
| 2026-59/23 | *Psittacula krameri* | cloacal swab | ***Positive*** | duck adenovirus 1 | *Barthadenovirus* | DAdV-1/Italy/*Psittacula_krameri*/2026-59/2023 | PP665610 |
| 2026-60/23 | *Cyanoramphus novaezelandiae* | cloacal swab | ***Positive*** | psittacine adenovirus 2 | *Siadenovirus* | PsAdV-2/Italy/*Cyanoramphus_novaezelandiae*/2026-60/2023 | PP665654 |
| 2026-61/23 | *Cyanoramphus novaezelandiae* | cloacal swab | ***Positive*** | psittacine adenovirus 2 | *Siadenovirus* | PsAdV-2/Italy/*Cyanoramphus_novaezelandiae*/2026-61/2023 | PP665655 |
| 2026-62/23 | *Cyanoramphus novaezelandiae* | cloacal swab | ***Positive*** | psittacine adenovirus 2 | *Siadenovirus* | PsAdV-2/Italy/*Cyanoramphus_novaezelandiae*/2026-62/2023 | PP665656 |
| 2026-63/23 | *Cyanoramphus novaezelandiae* | cloacal swab | ***Positive*** | psittacine adenovirus 2 | *Siadenovirus* | PsAdV-2/Italy/*Cyanoramphus_novaezelandiae*/2026-63/2023 | PP665657 |
| 2026-64/23 | *Cyanoramphus novaezelandiae* | cloacal swab | ***Positive*** | psittacine adenovirus 2 | *Siadenovirus* | PsAdV-2/Italy/*Cyanoramphus_novaezelandiae*/2026-64/2023 | PP665658 |
| 2026-65/23 | *Cyanoramphus novaezelandiae* | cloacal swab | ***Positive*** | psittacine adenovirus 2 | *Siadenovirus* | PsAdV-2/Italy/*Cyanoramphus_novaezelandiae*/2026-65/2023 | PP665659 |
| 2026-66/23 | *Cyanoramphus novaezelandiae* | cloacal swab | Negative | --- | --- | --- | --- |
| 2026-67/23 | *Cyanoramphus novaezelandiae* | cloacal swab | Negative | --- | --- | --- | --- |
| 2026-68/23 | *Cyanoramphus novaezelandiae* | cloacal swab | ***Positive*** | psittacine adenovirus 2 | *Siadenovirus* | PsAdV-2/Italy/*Cyanoramphus_novaezelandiae*/2026-68/2023 | PP665660 |
| 2026-69/23 | *Cyanoramphus novaezelandiae* | cloacal swab | ***Positive*** | duck adenovirus 1 | *Barthadenovirus* | DAdV-1/Italy/*Cyanoramphus_novaezelandiae*/2026-69/2023 | PP665611 |
| 2026-70/23 | *Cyanoramphus novaezelandiae* | cloacal swab | ***Positive*** | psittacine adenovirus 2 | *Siadenovirus* | PsAdV-2/Italy/*Cyanoramphus_novaezelandiae*/2026-70/2023 | PP665661 |
| 2026-71/23 | *Cyanoramphus novaezelandiae* | cloacal swab | ***Positive*** | psittacine adenovirus 2 | *Siadenovirus* | PsAdV-2/Italy/*Cyanoramphus_novaezelandiae*/2026-71/2023 | PP665662 |
| 2026-72/23 | *Psephotellus dissimilis* | cloacal swab | ***Positive*** | psittacine adenovirus 2 | *Siadenovirus* | PsAdV-2/Italy/*Psephotellus_dissimilis*/2026-72/2023 | PP665663 |
| 2026-73/23 | *Psephotellus dissimilis* | cloacal swab | ***Positive*** | psittacine adenovirus 2 | *Siadenovirus* | PsAdV-2/Italy/*Psephotellus_dissimilis*/2026-73/2023 | PP665664 |
| 2026-74/23 | *Psephotellus dissimilis* | cloacal swab | ***Positive*** | psittacine adenovirus 2 | *Siadenovirus* | PsAdV-2/Italy/*Psephotellus_dissimilis*/2026-74/2023 | PP665665 |
| 2026-75/23 | *Cyanoramphus novaezelandiae* | cloacal swab | ***Positive*** | psittacine adenovirus 2 | *Siadenovirus* | PsAdV-2/Italy/*Cyanoramphus_novaezelandiae*/2026-75/2023 | PP665666 |
| 2026-76/23 | *Cyanoramphus novaezelandiae* | cloacal swab | ***Positive*** | psittacine adenovirus 2 | *Siadenovirus* | PsAdV-2/Italy/*Cyanoramphus_novaezelandiae*/2026-76/2023 | PP665667 |
| 2026-77/23 | *Psephotus haematonotus* | cloacal swab | ***Positive*** | N.C. | *Siadenovirus* | AdV/Italy/*Psephotus_haematonotus*/2026-77/2023 | PP665675 |
| 2026-78/23 | *Psephotus haematonotus* | cloacal swab | ***Positive*** | N.C. | *Siadenovirus* | AdV/Italy/*Psephotus_haematonotus*/2026-78/2023 | PP665676 |
| 2026-79/23 | *Psephotus haematonotus* | cloacal swab | ***Positive*** | psittacine adenovirus 2 | *Siadenovirus* | PsAdV-2/Italy/*Psephotus_haematonotus*/2026-79/2023 | PP665668 |
| 2026-80/23 | *Psephotus haematonotus* | cloacal swab | Negative | --- | --- | --- | --- |
| 2026-81/23 | *Cyanoramphus novaezelandiae* | liver | Negative | --- | --- | --- | --- |
| 2026-82/23 | *Cyanoramphus novaezelandiae* | liver | Negative | --- | --- | --- | --- |
| 2026-83/23 | *Psephotellus dissimilis* | liver | Negative | --- | --- | --- | --- |
| 2026-84/23 | *Neopsephotus bourkii* | liver | ***Positive*** | N.C. | *Siadenovirus* | AdV/Italy/*Neopsephotus_bourkii*/2026-84/2023 | PP665677 |
| 2026-85/23 | *Neopsephotus bourkii* | liver | ***Positive*** | psittacine adenovirus 2 | *Siadenovirus* | PsAdV-2/Italy/*Neopsephotus_bourkii*/2026-85/2023 | PP665669 |
| 2026-86/23 | *Cyanoramphus novaezelandiae* | liver | ***Positive*** | N.C. | *Barthadenovirus* | AdV/Italy/*Cyanoramphus_novaezelandiae*/2026-86/2023 | PP665613 |
| 2026-87/23 | *Cyanoramphus novaezelandiae* | liver | ***Positive*** | N.C. | *Barthadenovirus* | AdV/Italy/*Cyanoramphus_novaezelandiae*/2026-87/2023 | PP665614 |
| 2026-88/23 | *Cyanoramphus novaezelandiae* | liver | ***Positive*** | psittacine adenovirus 2 | *Siadenovirus* | PsAdV-2/Italy/*Cyanoramphus_novaezelandiae*/2026-88/2023 | PP665670 |
| 2026-89/23 | *Agapornis roseicollis* | liver | ***Positive*** | duck adenovirus 1 | *Barthadenovirus* | DAdV-1/Italy/*Agapornis_roseicollis*/2026-89/2023 | PP665612 |
| 2026-90/23 | *Agapornis roseicollis* | liver | Negative | --- | --- | --- | --- |
| 2026-91/23 | *Cyanoramphus novaezelandiae* | liver | Negative | --- | --- | --- | --- |
| 2026-92/23 | *Cyanoramphus novaezelandiae* | liver | ***Positive*** | psittacine adenovirus 2 | *Siadenovirus* | PsAdV-2/Italy/*Cyanoramphus_novaezelandiae*/2026-92/2023 | PP665671 |
| 2026-93/23 | *Cyanoramphus novaezelandiae* | liver | ***Positive*** | psittacine adenovirus 2 | *Siadenovirus* | PsAdV-2/Italy/*Cyanoramphus_novaezelandiae*/2026-93/2023 | PP665672 |
| 2026-94/23 | *Cyanoramphus novaezelandiae* | liver | ***Positive*** | N.C. | *Aviadenovirus* | AdV/Italy/*Cyanoramphus_novaezelandiae*/2026-94/2023 | PP665615 |
| 2026-95/23 | *Psittacula krameri* | liver | Negative | --- | --- | --- | --- |

*N.C. = Not Classified*
