# Supplementary material for: High prevalence and genetic heterogeneity of adenoviruses at a psittacine breeding facility
Source: Vet Res Commun. 2024 Sep 12;48(6):4113–22. doi: 10.1007/s11259-024-10533-7 (PMC11538262; doi:10.1007/s11259-024-10533-7)
Supplement: Supplementary file 2 — Supplementary Material 2 [file 11259_2024_10533_MOESM2_ESM.docx]

**High prevalence and genetic heterogeneity of adenoviruses at a psittacine breeding facility**

**Veterinary Research Communications**

Gabriele Lizzi^1^, Simone Fasana^2^, Guido Grilli^2^, Giulia Quaglia^1^, Sara Pedrazzoli^1^, Giulia Graziosi^1^, Elena Catelli^1^, Laura Musa^2^, Maria Cristina Rapi^2^, Caterina Lupini^1^

^1^ Department of Veterinary Medical Sciences, University of Bologna, Via Tolara di Sopra 50, Ozzano dell'Emilia (BO), 40064, Italy

^2^ Department of Veterinary Medicine and Animal Science, University of Milan, Via dell'Università 6, Lodi, 26900, Italy

**Corresponding author:**

Gabriele Lizzi, Department of Veterinary Medical Sciences, University of Bologna, Via Tolara di Sopra 50, Ozzano dell'Emilia (BO), 40064, Italy. Email: gabriele.lizzi2@unibo.it

***Table Supplementary 2****. Details of the pol gene sequences retrieved from GenBank which were used for the phylogenetic analysis.*

| **Adenovirus strain** | **Country** | **Psittacine host** | **Genus** | **Type** | **Accession** | **Reference** |
| --- | --- | --- | --- | --- | --- | --- |
| CSPAdV-2 | Antarctica | *Pygoscelis antarcticus* | *Siadenovirus* | chinstrap penguin adenovirus 2 | KP144329 | Lee et al. (2016) |
| IDL20-0871 | USA | *Uria aalge* | *Siadenovirus* | common murre adenovirus | MN480433 | - |
| DAdV-1 | Germany | - | *Barthadenovirus* | duck adenovirus 1 | AC_000004 | Hess et al. (1997) |
| GD-2019 | China | *Cairina moschata* | *Aviadenovirus* | duck adenovirus 4 | MN733730 | - |
| Isolate B | Australia | *Poephila acuticauda* | *Barthadenovirus* | Estrildidae adenovirus 2 | MK413651 | Phalen et al. (2019) |
| - | - | *-* | *Aviadenovirus* | fowl adenovirus 1 | AC_000014 | Davison et al. (2003) |
| KR5 | - | *-* | *Aviadenovirus* | fowl adenovirus 4 | HE608152 | Marek et al. (2012) |
| Strain 340 | Northern Ireland | *Gallus gallus* | *Aviadenovirus* | fowl adenovirus 5 | KC493646 | Marek et al. (2013) |
| CR119 | Japan | *Gallus gallus* | *Aviadenovirus* | fowl adenovirus 6 | KT862808 | Marek et al. (2016) |
| A-2A | - | *-* | *Aviadenovirus* | fowl adenovirus 9 | AF083975 | Cao et al. (1998) |
| Isolate A | Australia | *Erythrura gouldiae* | *Siadenovirus* | Gouldian finch adenovirus 1 | MK413653 | Phalen et al. (2019) |
| 5957/SZ | Hungary | *Parus major* | *Siadenovirus* | great tit adenovirus 1 | FJ849795 | Kovács et al. (2010) |
| S478/20 | Germany | *Parus major* | *Siadenovirus* | great tit adenovirus 3 | MW508338 | Fischer et al. (2021) |
| - | Japan | *Bubo bengalensis* | *Aviadenovirus* | owl adenovirus | LC638697 | Kobayashi et al. (2022) |
| AU2787 | Australia | *Acanthorhynchus tenuirostris* | *Barthadenovirus* | passerine adenovirus 1 | MT674683 | Athukorala et al. (2020) |
| M621 | Hungary | *Columba livia domestica* | *Siadenovirus* | pigeon adenovirus 5 | KX555532 | Ballmann and Harrach (2016) |
| 18VIR149_ITA_2018 | Italy | *Poicephalus senegalus* | *Aviadenovirus* | psittacine adenovirus 1 | MH580295 | Milani et al. (2018) |
| WHC1025 | Australia | *Trichoglossus haematodus* | *Aviadenovirus* | psittacine adenovirus 1 | MN238644 | Vaz et al. (2020) |
| OBP2209 | Australia | *Neophema chrysogaster* | *Siadenovirus* | psittacine adenovirus 2 | MW365934 | Athukorala et al. (2021) |
| Isolate 7 | Romania | *Cyanoramphus novaezelandiae* | *Siadenovirus* | psittacine adenovirus 2 | KC248187 | Ballmann and Vidovszky (2013) |
| Isolate 70702 | Germany | *Cyanoramphus novaezelandiae* | *Siadenovirus* | psittacine adenovirus 2 | OM994429 | Konicek et al. (2022) |
| PsAdV2/S10/AUS | Australia | *Neophema splendida* | *Siadenovirus* | psittacine adenovirus 2 | MZ364296 | Sarker (2021) |
| WVL19065-01E | USA | *Psittacus erithacus* | *Siadenovirus* | psittacine adenovirus 2 | MZ562791 | Surphlis et al. (2022) |
| FL-DAK | USA | *Psittacula cyanocephala* | *Siadenovirus* | psittacine adenovirus 2 | EU056825 | Wellehan et al. (2009) |
| BR_DF | Brazil | *Psittacara leucophthalmus* | *Barthadenovirus* | psittacine adenovirus 3 | MN025529 | Duarte et al. (2019) |
| HKU/Parrot19 | Hong Kong | *Amazona farinosa* | *Barthadenovirus* | psittacine adenovirus 3 | KJ675568 | To et al. (2014) |
| CS15-4016 | Australia | *Poicephalus rufiventris* | *Aviadenovirus* | psittacine adenovirus 4 | KX577802 | Das et al. (2017) |
| 142878ffpeb | USA | *Aratinga solstitialis* | *Siadenovirus* | psittacine adenovirus 5 | MN450070 | Gottdenker et al. (2019) |
| MA41 | Hungary | *Aratinga nenday* | *Siadenovirus* | psittacine adenovirus 5 | OL603908 | Harrach et al. (2023) |
| 129AM | Slovenia | *Melopsittacus undulatus* | *Siadenovirus* | psittacine adenovirus 5 | OK058275 | Zadravec et al. (2022) |
| BrdKdnyDNA | Australia | *Melopsittacus undulatus* | *Siadenovirus* | psittacine adenovirus 6 | MN687905 | - |
| CorAdV1/Melbourne/2015 | Australia | *Cacatua sanguinea* | *Siadenovirus* | psittacine adenovirus 7 | MK227353 | Sutherland et al. (2019) |
| Meyer’s parrot | USA | *Poicephalus meyeri* | *Aviadenovirus* | psittacine adenovirus 8 | AY644731 | Wellehan et al. (2005) |
| AL32 | Slovenia | *Psittacus erithacus* | *Aviadenovirus* | psittacine adenovirus 8 | OK058272 | Zadravec et al. (2022) |
| AL87 | Slovenia | *Nymphicus hollandicus* | *Barthadenovirus* | psittacine adenovirus 9 | OK058273 | Zadravec et al. (2022) |
| 22AM | Slovenia | *Psittacula krameri* | *Barthadenovirus* | psittacine adenovirus 10 | OK058274 | Zadravec et al. (2022) |
| par083ade1 | China | *Ara glaucogularis* | *Barthadenovirus* | psittacine adenovirus 11 | MT138098 | - |
| LBF_44 | USA | *Agapornis roseicollis* | *Aviadenovirus* | psittacine adenovirus 12 | PP555268 | - |
| wwb174ade01 | China | *Phylloscopus schwarzi* | *Barthadenovirus* | Radde's warbler adenovirus 1 | MT138103 | - |
| - | United Kingdom | *Parabuteo unicinctus* | *Siadenovirus* | raptor adenovirus 1 | EU715130 | Kovács and Benkö (2009) |
| T03 | Antarctica | *Stercorarius maccormicki* | *Siadenovirus* | South Polar skua adenovirus 1 | HM585353 | Park et al. (2012) |
| SIB_81 | Russia | *Sterna hirundo* | *Barthadenovirus* | tern atadenovirus 1 | OL692338 | Matsvay et al. (2021) |
| D90/2 | Hungary | *Meleagris gallopavo* | *Aviadenovirus* | turkey adenovirus 1 | GU936707 | Kaján et al. (2010) |
| - | USA | *Meleagris gallopavo* | *Siadenovirus* | turkey adenovirus 3 | AF074946 | Pitcovski et al. (1998) |
| TNI1 | - | *Meleagris gallopavo* | *Aviadenovirus* | turkey adenovirus 4 | KF477312 | Marek et al. (2014) |
| 1277BT | - | *Meleagris gallopavo* | *Aviadenovirus* | turkey adenovirus 5 | KF477313 | Marek et al. (2014) |
| Strain 37869 | Germany | *Ploceus vitellinus* | *Barthadenovirus* | vitelline masked weaver adenovirus 1 | MN380538 | Rinder et al. (2020) |
| BR_DF2 | Brazil | *Psittacara leucophthalmus* | *Aviadenovirus* | white-eyed parakeet 2 | MN153802 | Duarte et al. (2019) |
| WHC1081 | Australia | *Ptilotula penicillata* | *Barthadenovirus* | white-plumed honeyeater adenovirus 1 | MN238667 | Vaz et al. (2020) |
| warbler203 | China | *Phylloscopus inornatus* | *Barthadenovirus* | yellow-browed warbler adenovirus 1 | MT138101 | - |
| Strain 47535 | Germany | *Taeniopygia guttata* | *Siadenovirus* | zebra finch adenovirus 2 | MN380549 | Rinder et al. (2020) |

**REFERENCES**

1. Athukorala A, Forwood JK, Phalen DN, Sarker S (2020) Molecular characterisation of a novel and highly divergent passerine adenovirus 1. Viruses 12(9):1036. <https://doi.org/10.3390/v12091036>
2. Athukorala A, Phalen DN, Das A, Helbig KJ, Forwood JK, Sarker S (2021) Genomic characterisation of a highly divergent *Siadenovirus* (psittacine siadenovirus F) from the critically endangered orange-bellied parrot (*Neophema chrysogaster*). Viruses 13(9):1714. <https://doi.org/10.3390/v13091714>
3. Ballmann MZ, Harrach B (2016) Detection and partial genetic characterisation of novel avi- and siadenoviruses in racing and fancy pigeons (*Columba livia domestica*). Acta Vet. Hung. 64(4):514-528. <https://doi.org/10.1556/004.2016.047>
4. Ballmann MZ, Vidovszky MZ (2013) Detection of broad-host-range psittacine adenovirus (PsAdV-2) in representatives of different parrot species. Magy Allatorvosok Lapja 135 (2):78-84
5. Benkő M, Arnberg N, Heim A, Hess M, Haján GL, Kajon A, Kuhn JH, Mittal SK, Podgorski II, Postler TS, San Martín C, Wadell G, Watanabe H, Vidovszky MZ, Harrach B (2023) Rename genus *Atadenovirus* and add seven new species; create seven new species in genus *Aviadenovirus*, five new species in genus *Mastadenovirus*, three new species in genus *Siadenovirus*; and rename 86 adenovirid species (Rowavirales: *Adenoviridae*). <https://ictv.global/filebrowser/download/15312>
6. Cao JX, Krell PJ, Nagy E (1998) Sequence and transcriptional analysis of terminal regions of the fowl adenovirus type 8 genome. J Gen Virol. 79 (10):2507-2516. <https://doi.org/10.1099/0022-1317-79-10-2507>
7. Das S, Fearnside K, Sarker S, Forwood JK, Raidal SR (2017) A novel pathogenic aviadenovirus from red-bellied parrots (*Poicephalus rufiventris*) unveils deep recombination events among avian host lineages. Virology 502:188-197. https://doi.org/10.1016/j.virol.2016.12.031
8. Davison AJ, Benkő M, Harrach B (2003) Genetic content and evolution of adenoviruses. J Gen Virol. 84(11):2895-2908. <https://doi.org/10.1099/vir.0.19497-0>
9. Duarte MA, Silva JMF, Brito CR, Teixeira DS, Melo FL, Ribeiro BM, Nagata T, Campos FS (2019) Faecal virome analysis of wild animals from Brazil. Viruses 11(9):803. <https://doi.org/10.3390/v11090803>
10. Fischer L, Peters M, Merbach S, Eydner M, Kuczka A, Lambertz J, Kummerfeld M, Kahnt K, Weiss A, Petersen H (2021) Increased mortality in wild tits in North Rhine-Westphalia (Germany) in 2020 with a special focus on *Suttonella ornithocola* and other infectious pathogens. Eur J Wildl Res 67:56. https://doi.org/10.1007/s10344-021-01500-7
11. Gottdenker NL, Gregory CR, Ard MB, Lorenz WW, Nilsen RA, Ritchie BW (2019) Histopathologic changes, ultrastructure, and molecular characterization of an adenovirus in a sun conure (*Aratinga solstitialis*). Avian Dis. 63(3):531-538. https://doi.org/10.1637/aviandiseases-D-19-00080
12. Harrach B, Megyeri A, Papp T, Ursu K, Boldogh SA, Kaján GL (2023) A screening of wild bird samples enhances our knowledge about the biodiversity of avian adenoviruses. Vet Res Commun 47(1):297-303. https://doi.org/10.1007/s11259-022-09931-6
13. Hess M, Blöcker H, Brandt P (1997) The complete nucleotide sequence of the egg drop syndrome virus: an intermediate between mastadenoviruses and aviadenoviruses. Virology 238(1):145-156. https://doi.org/10.1006/viro.1997.8815
14. Kaján GL, Stefancsik R, Ursu K, Palya V, Benkő M (2010) The first complete genome sequence of a non-chicken aviadenovirus, proposed to be turkey adenovirus 1. Virus Res. 153(2):226-233. <https://doi.org/10.1016/j.virusres.2010.08.006>
15. Kobayashi H, Uchida Y, Fujino K, Horie M, Umezawa E, Aihara N, Kamiie J, Shimoda H, Maeda K, Une Y, Taharaguchi S (2022) Isolation and whole-genome sequencing of a novel aviadenovirus from owls in Japan. Arch Virol 167:829–838. https://doi.org/10.1007/s00705-022-05380-3
16. Konicek C, Heenemann K, Cramer K, Vahlenkamp TW, Schmidt V (2022) Case series of disseminated xanthogranulomatosis in red-crowned parakeets (*Cyanoramphus novaezelandiae*) with detection of psittacine adenovirus 2 (PsAdV-2). Animals (Basel) 12(18):2316. <https://doi.org/10.3390/ani12182316>
17. Kovács ER, Benkő M (2009) Confirmation of a novel siadenovirus species detected in raptors: partial sequence and phylogenetic analysis. Virus Res. 140(1-2):64-70. <https://doi.org/10.1016/j.virusres.2008.11.005>
18. Kovács ER, Jánoska M, Dán A, Harrach B, Benkő M (2010) Recognition and partial genome characterization by non-specific DNA amplification and PCR of a new siadenovirus species in a sample originating from *Parus major*, a great tit. J Virol Methods. 163(2):262-268. <https://doi.org/10.1016/j.jviromet.2009.10.007>
19. Lee S-Y, Kim J-H, Seo T-K, No J-S, Kim H, Kim W-K, Choi H-G, Kang S-H, Song J-W (2016) Genetic and molecular epidemiological characterization of a novel adenovirus in Antarctic penguins collected between 2008 and 2013. PLoS One 11(6):e0157032. <https://doi.org/10.1371/journal.pone.0157032>
20. Marek A, Ballmann MZ, Kosiol C, Harrach B, Schlötterer C, Hess M (2014) Whole-genome sequences of two turkey adenovirus types reveal the existence of two unknown lineages that merit the establishment of novel species within the genus *Aviadenovirus*. J Gen Virol. 95(1):156-170. https://doi.org/10.1099/vir.0.057711-0
21. Marek A, Kaján GL, Kosiol C, Benkő M, Schachner A, Hess M (2016) Genetic diversity of species Fowl aviadenovirus D and Fowl aviadenovirus E. J Gen Virol. 97(9):2323-2332. <https://doi.org/10.1099/jgv.0.000519>
22. Marek A, Kosiol C, Harrach B, Kaján GL, Schlötterer C, Hess M (2013) The first whole genome sequence of a Fowl adenovirus B strain enables interspecies comparisons within the genus *Aviadenovirus*. Vet Microbiol. 166(1-2):250-256. <https://doi.org/10.1016/j.vetmic.2013.05.017>
23. Marek A, Nolte V, Schachner A, Berger E, Schlötterer C, Hess M (2012) Two fiber genes of nearly equal lengths are a common and distinctive feature of Fowl adenovirus C members. Vet Microbiol. 156(3-4):411-417. <https://doi.org/10.1016/j.vetmic.2011.11.003>
24. Matsvay A, Dyachkova M, Mikhaylov I, Kiselev D, Say A, Burskaia V, Artyushin I, Khafizov K, Shipulin G (2021) Complete genome sequence, molecular characterization and phylogenetic relationships of a novel tern *Atadenovirus*. Microorganisms 10(1):31. <https://doi.org/10.3390/microorganisms10010031>
25. Milani A, Zamperin G, Fusaro A, Salviato A, Bano L, Zandonà L, Brunetta R, Monne I (2018) Complete genome sequence of psittacine adenovirus 1, identified from *Poicephalus senegalus* in Italy. Microbiol Resour Announc 7(11):e01037-18. https://doi.org/10.1128/mra.01037-18
26. Park YM, Kim JH, Gu SH, Lee SY, Lee MG, Kang YK, Kang SH, Kim HJ, Song JW (2012) Full genome analysis of a novel adenovirus from the South Polar skua (*Catharacta maccormicki*) in Antarctica. Virology 422(1):144-150. <https://doi.org/10.1016/j.virol.2011.10.008>
27. Pitcovski J, Mualem M, Rei-Koren Z, Krispel S, Shmueli E, Peretz Y, Gutter B, Gallili GE, Michael A, Goldberg D (1998) The complete DNA sequence and genome organization of the avian adenovirus, hemorrhagic enteritis virus. Virology 249(2):307-315. <https://doi.org/10.1006/viro.1998.9336>
28. Phalen DN, Agius J, Vaz FF, Eden JS, Setyo LC, Donahoe S (2019) A survey of a mixed species aviary provides new insights into the pathogenicity, diversity, evolution, host range, and distribution of psittacine and passerine adenoviruses. Avian Pathol 48(5):437-443. https://doi.org/10.1080/03079457.2019.1617835
29. Rinder M, Schmitz A, Baas N, Korbel R (2020) Molecular identification of novel and genetically diverse adenoviruses in Passeriform birds. Virus Genes 56(3):316-324. https://doi.org/10.1007/s11262-020-01739-3
30. Sarker S (2021) Metagenomic detection and characterisation of multiple viruses in apparently healthy Australian *Neophema* birds. Sci Rep 11(1):20915. https://doi.org/10.1038/s41598-021-00440-1
31. Surphlis AC, Dill-Okubo JA, Harrach B, Waltzek T, Subramaniam K (2022) Genomic characterization of psittacine adenovirus 2, a *Siadenovirus* identified in a moribund African grey parrot (*Psittacus erithacus*). Arch Virol 167(3):911-916. https://doi.org/10.1007/s00705-021-05341-2
32. Sutherland M, Sarker S, Vaz PK, Legione AR, Devlin JM, Macwhirter PL, Whiteley PL, Raidal SR (2019) Disease surveillance in wild Victorian cacatuids reveals co-infection with multiple agents and detection of novel avian viruses. Vet Microbiol 235:257-264. https://doi.org/10.1016/j.vetmic.2019.07.012
33. To KK, Tse H, Chan WM, Choi GK, Zhang AJ, Sridhar S, Wong SC, Chan JF, Chan AS, Woo PC, Lau SK, Lo JY, Chan KH, Cheng VC, Yuen KY (2014) A novel psittacine adenovirus identified during an outbreak of avian chlamydiosis and human psittacosis: zoonosis associated with virus-bacterium coinfection in birds. PLoS Negl Trop Dis 8(12):e3318. https://doi.org/10.1371/journal.pntd.0003318
34. Vaz FF, Raso TF, Agius JE, Hunt T, Leishman A, Eden JS, Phalen DN (2020) Opportunistic sampling of wild native and invasive birds reveals a rich diversity of adenoviruses in Australia. Virus Evol 6(1):veaa024. https://doi.org/10.1093/ve/veaa024
35. Wellehan JFX, Greenacre CB, Fleming GJ, Stetter MD, Childress AL, Terrell SP (2009) *Siadenovirus* infection in two psittacine bird species. Avian Pathol 38(5):413-417. https://doi.org/10.1080/03079450903183660
36. Wellehan JFX, Johnson AJ, Latimer KS, Bischoff K, Lafortune M, Jacobson ER (2005) Identification and initial characterization of an adenovirus associated with fatal hepatic and lymphoid necrosis in a Meyer's parrot (*Poicephalus meyeri*). J Avian Med Surg 19(3):191-197 <https://doi.org/10.1647/2004-003.1>
37. Zadravec M, Račnik J, Slavec B, Ballmann MZ, Kaján GL, Doszpoly A, Zorman-Rojs O, Marhold C, Harrach B (2022) Novel adenoviruses from captive psittacine birds in Slovenia. Comp Immunol Microbiol Infect Dis 90–91:101902. <https://doi.org/10.1016/j.cimid.2022.101902>
